# Ginseng Prong Added to Broiler Diets Reduces Lipid Peroxidation in Refrigerated and Frozen Stored Poultry Meats

**DOI:** 10.3390/molecules26134033

**Published:** 2021-07-01

**Authors:** Melody M. C. Lai, Huiying Amelie Zhang, David D. Kitts

**Affiliations:** Food Nutrition and Health Program, Faculty of Land and Food Systems, University of British Columbia, Vancouver, BC V6T 1Z4, Canada; melody.lai@canada.ca (M.M.C.L.); amelie.huiying.zhang@ubc.ca (H.A.Z.)

**Keywords:** antioxidant, peroxides, malondialdehyde, shelf-life, ginseng

## Abstract

Fatty acid content and lipid oxidation products were compared in chicken breast and leg meats derived from birds fed on animal-fat- and vegetable-oil-based diets, supplemented with ginseng prong powder. The first experiment examined polyunsaturated fatty acid (PUFA) content and the formation of primary and secondary lipid oxidation products in meats stored at refrigeration temperatures (4 °C) for up to 10 days, while the second experiment examined similar changes in the poultry meats when frozen stored at −18 °C, for up to six months. Results showed that initial lipid hydroperoxide concentrations increased in both breast and leg meat within the first week of refrigerated storage and also was ongoing during the first three to four months of frozen storage. A higher (*p* < 0.05) PUFA content in leg meat, especially in broilers fed a vegetable-oil-blended diet, corresponded to greater tendency for generation of primary lipid oxidation products after refrigerated and frozen storage (*p* < 0.05). The inclusion of powdered ginseng prong in broiler diets significantly inhibited (*p* < 0.05) secondary lipid oxidation products (e.g., malonaldehyde [MDA]) formation in both stored leg and breast meat, compared to controls. Significant interactions (*p* < 0.05) were obtained for storage time and inclusion of ginseng against production of primary and secondary lipid oxidation in broiler breast and leg meats from broilers fed PUFA-containing diets. We conclude that including ginseng prong in broiler growing diets represents a viable strategy to control lipid oxidation in refrigerated/cold-stored meat products.

## 1. Introduction

The addition of fats and oils to poultry rations is to increase the energy content of the diet. PUFA rich fats have a relatively higher digestibility than saturated fats and are better deposited in muscle fat, due to higher incorporated into phospholipids, which in turn are present in a higher proportion in muscle compared to adipose [1]. Rations containing PUFA rich fats are however highly vulnerable to oxidation reactions during storage. Dietary fat sources that have been used in broiler rations and which vary in the degree of unsaturation (e.g., saturated fat versus sunflower versus olive oils, respectively) and in the presence of alpha-tocopherol, and beta-carotene have been reported to have different effects on the onset of lipid oxidation in broiler meats [2]. A cascade of chemical reactions that lead to breakdown products derived from hydroperoxides to secondary lipid oxidation products, notably aldehydes and ketones, ultimately lowers sensory, nutritional and shelf-life quality [3]. Poultry meats derived from broilers fed a blend of antioxidants, e.g., propyl gallate and ethoxyquin, have showed reduced lipid oxidation and drip loss in final poultry products [4]. Baghban Kanani et al. [5] measured hydroperoxide and thiobarbituric acid (TBA) values and reported that lipid oxidation could be reduced in meats when broilers were fed diets supplemented with turmeric and cinnamon powders. Quercetin, with recognized antioxidant activity, has also been used to stabilize chicken pates [6]. Indeed, stabilizing dietary lipid sources by inclusion of other known antioxidants in the formulation has also achieved success with vitamin C [7], selenium [8], rosemary [9], and sage [10]. This practice could also lead to reducing the intake of oxidized feed by poultry [11].

**Figure 1:**
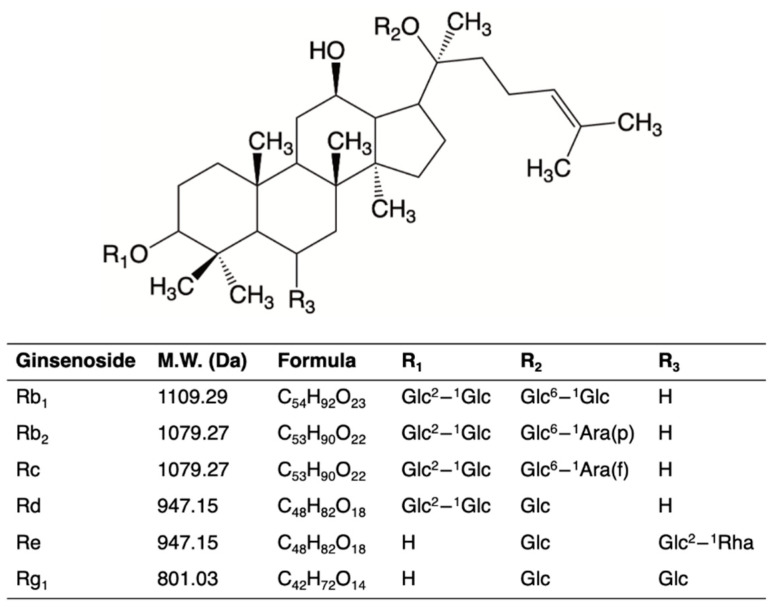
Chemical structure of major ginsenosides in ginseng [27]. Glc, β-D-glucose; Ara(p), α-L-arabinopyranose; Ara(f), α-L-arabinofuranose; Rha, α-L-rhamnose.

The ginseng plant is a traditional Chinese herb grown in North America, composed of plant parts such as an aerial stem bearing leaves and a root system that consists of the primary root and fibers called prongs. Commercial preparation of the root involves removing prongs, which can be powdered for alternative usage. Numerous biologically active components exist in ginseng root, leaf steam and prong. These include ginsenosides (Figure 1), flavonoids, triterpenoids and polysaccharides [12,13,14]. A number of studies have shown antioxidant activity of ginseng derived from both *Panax quinefolium*, or *Panax ginseng* C.A. Meyer sources, to include free radical scavenging activity towards hydroxyl and oxygen radicals and affinity to prevent Fenton reaction induced peroxidation, using both lipid microsomes and human low-density lipoprotein (LDL) model systems [15,16,17,18]. Liu et al. [19] used an in vitro erythrocyte hemolysis model to compare relative antioxidant activity of different ginsenosides and showed ginsenosides with greater hydrophilicity (e.g., Rc and Rb_1_) had relatively greater antioxidant activity. Feeding ginseng has also evoked in vivo shifts in antioxidant enzyme activities, including superoxide dismutase and catalase [20,21] and earlier work also reported an affinity for ginseng to enhance hepatic antioxidant activity that resulted in lower lipid peroxidation [22]. In addition, ginseng has been shown to be relevant to lipid metabolism and lipid acquisition using cultured adipocytes [23] and by monitoring plasma lipid responses in ginseng-fed rodents that were fed on a high fat diet [24]. Positive effects of ginseng consumption on adipose tissue metabolism, associated with oxidative status has also been shown in humans [25,26]. Taken together, the bioactive properties of ginseng suggest a possible role to reduce susceptibility lipid peroxidation reactions in refrigerated meat products if included in broiler diets throughout their life cycle.

The objective of the present study was to conduct a thorough analysis of the effects of animal fat versus vegetable oil diets, fed in a commercial setting, on the susceptibility of broiler meats to lipid peroxidation during refrigeration and freezing storage. We further tested the efficacy of ginseng supplementation to formulated diets to mitigate lipid peroxidation in stored breast and leg meats.

## 2. Results

Inclusion of ginseng prong at both levels in the animal-fat- and vegetable-oil-based diets, respectively, did not produce a significant difference in final body weight of broilers compared to counterparts fed on control diets (data not shown). A comparison of both leg and breast meat quality parameters from birds fed the experimental diets is given in Appendix A Table A2. For the most part, quality parameters did not differ significantly between different dietary treatments.

### 2.1. Experiment 1: Short-Term Refrigeration Storage

#### 2.1.1. Crude Lipid Analysis

Initial crude lipid levels were essentially 2.5- to 3.5-fold higher in leg compared to breast meat (Table 1). Total crude lipid content in breast meats increased (*p* < 0.05) with ginseng feeding, while refrigeration storage produced a less defined change in total crude lipid content. Only a single factor, i.e., feeding ginseng at higher concentration, increased (*p* < 0.05) crude lipid in the leg meats derived from broilers fed on the animal fat diet. There were no significant treatment interactions associated with ginseng feeding and storage time in both breast and leg meats derived from birds fed on either the animal fat- or vegetable oil- blended diets, respectively.

#### 2.1.2. Fatty Acid Profiles

Broilers fed the vegetable-oil-based diet produced meat products that were more prone to losses in total PUFAs during refrigerated storage, compared to the meats derived from birds fed on diets containing animal fat (Table 2). There were no significant changes in either total saturated or monounsaturated fatty acids in both breast and leg meats due to diet fat type, the refrigeration storage time or presence of ginseng (data not shown). The presence of ginseng in animal fat- based diets enabled preservation of total PUFAs in both breast and leg meats, whereas reductions were observed in samples derived from birds fed vegetable-oil-based diets. A significant (*p* = 0.03) interaction was obtained with storage time and presence of ginseng for breast meats derived from vegetable-oil-based diets, but not for leg meat.

#### 2.1.3. Lipid Hydroperoxide Analysis

In general, broilers fed on the vegetable-oil-based diets exhibited higher lipid hydroperoxide concentrations that counterparts fed on animal-fat-based diets (Table 3). Hydroperoxide content was also greater and appeared earlier in refrigerated storage in leg meats compared to breast meat (*p* < 0.05). Temporal concentrations of hydroperoxides in breast meats derived from birds fed on animal fat diets increased significantly (*p* < 0.05) throughout 10 days of refrigeration storage; however, those from birds fed on ginseng-supplemented vegetable oil diets showed a peak hydroperoxide content at day 4, which was followed by a significant decrease (*p* < 0.05), a finding which was not observed in the control. The increases in primary lipid peroxidation in leg meats during 10 days of refrigeration storage was greater compared to breast meats and reached comparatively higher concentrations in broilers fed on the vegetable-oil-based diet. This difference was related to the fact that the initial concentrations of lipid hydroperoxides were also lower in meat obtained from broilers fed on diets containing animal fat compared to vegetable oil. Only in the leg meats from birds fed on the vegetable oil were there significant (*p* < 0.05) decreases in lipid peroxidation products after 10 days refrigeration storage.

The inclusion of ginseng in vegetable-oil-based diets reduced the progression of primary lipid oxidation product accumulation (*p* < 0.05); especially during the first week storage in breast meats, relatively more so than that observed in leg meat. The relatively higher (*p* < 0.05) initial lipid peroxide concentrations measured in breast and leg meats derived from birds fed on the vegetable-oil-based diet was exasperated in birds fed on the higher level of ginseng supplementation. The effects of short-term refrigeration storage and the presence of ginseng in the poultry ration resulted in a significant interaction between storage time and ginseng feeding (*p* < 0.001) in both breast and leg meats, respectively, regardless of the dietary fat source.

#### 2.1.4. TBA Analysis

In general, changes in TBA values measured in both control breast and leg meats corresponded to the duration of refrigerated storage up to day 7, before typically declining; the decline in TBA values did not occur in the ginseng groups (Table 4). Changes in TBA values throughout storage corresponded to initial concentrations in both vegetable oil- and animal-fat-based diets. Significant (*p* < 0.05) increases in MDA, initially observed on day 2 in both meat samples, reached relatively higher concentrations by day 7. This was followed by a subsequent decrease (*p* < 0.05) in TBA value at day 10 of storage in breast meats derived from controls; this was not observed in the groups fed on diets with ginseng. Broilers fed the animal fat diets containing ginseng had a relatively small increase in MDA values initially, and absolute values were significantly (*p* < 0.05) lower than controls at the baseline and throughout the 10-day refrigerated storage period. This effect was also observed in counterparts fed the vegetable-oil-based diets. A two-way ANOVA analysis indicated a significant interaction (*p* < 0.001) for the duration of refrigeration and ginseng feeding in both breast and leg meats derived from birds fed on vegetable-oil- or animal-fat-derived diets.

### 2.2. Experiment 2: Long-Term Frozen Storage

Total crude lipid contents in poultry meats in Experiment 2 birds were almost three time greater in leg compared to breast meats and declined very little with extended frozen storage (Table 5). Extending the freezing time of meats up to 6 months resulted in small losses of crude lipid associated with both the leg and breast meats, from both animal and vegetable diets, respectively. There was no significant interaction between ginseng feeding and frozen storage time period for crude lipid content in both breast and leg meats.

Monitoring the change in lipid hydroperoxide content in both breast and leg meats over a 6-month period of frozen storage revealed that the practice of both feeding ginseng and the duration of frozen storage were significant factors that affected the extent of lipid oxidation (*p* < 0.05) (Table 6). Primary lipid oxidation products were relatively greater in leg compared to breast meat, as reported in Experiment 1 for meats that were refrigerated. Broiler breast and leg meats were predisposed to greater peroxidation during long-term frozen storage (*p* < 0.05) regardless of whether birds were fed vegetable oil- or animal fat- based diets. Peak lipid hydroperoxide contents were obtained at 4 months of frozen storage in both meat sources, whereas the peak in breast meats from ginseng supplementation occurred earlier at 2 months of storage. The lipid hydroperoxide level declined dramatically (*p* < 0.05) at 6 months of storage in all groups. A significant treatment interaction (*p* < 0.001) between ginseng feeding and frozen storage was observed for both breast and leg meats derived from broilers fed either vegetable oil or animal fats.

Breast and leg meats collected from broilers fed on the vegetable-oil-based diet con-tained similar MDA content compared to counterpart samples derived from birds fed on the animal fat diet (Table 7). This was observed prior to freezing and changes in MDA content followed a similar trend throughout the extended frozen storage period. The TBA values measured in both meat sources were significantly lower (*p* < 0.05) throughout the frozen storage period when meats were derived from birds fed on the ginseng diets. Fortifying vegetable oil diets with higher ginseng content resulted in lower (*p* < 0.05) TBA values in both breast and leg meat. The only exception was the apparent lack of any effect of ginseng to reduce TBA values in breast meat at 6 months of frozen storage from birds fed the animal-fat-based diet. A significant interaction between duration of frozen storage and the level of ginseng added to the diet on MDA concentration was significant in both breast and leg broiler meats (*p* < 0.01) (Table 7).

## 3. Discussion

This study compared the efficacy of adding a standardized ginsenoside powder derived from ginseng prong to broiler diets formulated using either an animal-fat-based or a vegetable-oil-based diet, respectively, for the purpose of reducing lipid oxidation in PUFA enhanced broiler meats during both refrigeration and prolonged frozen storage. Specifically, we assessed the anti-peroxidation capacity of ginseng in both breast and leg meats, respectively, in birds that were given diets that varied in the content of unsaturated fat.

Our study was designed to include ginseng in broiler diets as a potential antioxidant for the primary purpose to directly retain quality of broiler diets after slaughter and, subsequently, provide a carryover effect to retain quality of meat products during subsequent storage. The results may be influenced to some extent by a physiological/biochemical effect attributed to the bioactive properties of ginseng, previously reported to modulate whole-body lipid metabolism. For example, others reported ginseng to modulate both whole-body lipid metabolism as well as adipose lipid acquisition. Yokozawa et al. [28] achieved enhanced lipogenesis in rodents when applying intraperitoneal injection of a ginseng extract and in vitro cell culture studies have shown ginseng to stimulate lipid deposition in adipocytes [23]. Poultry meats at time zero in our study contained similar, or only small total crude lipid content differences in all dietary treatments for leg and breast, respectively, regardless of the inclusion of ginseng. This would indicate that changes in lipid content or susceptibility to peroxidation reported herein were related more to chemical reactions occurring during prolonged refrigeration or frozen storage than metabolic influences concerning lipid biosynthesis. Of interest is the finding that prolonged oral administration of a standardized Panax ginseng extract, that contained the same ginsenosides, Rb_1_, Rb_2_, Rc, Rd, Re and Rg_1_ present in our ginseng prong, was shown to protect against lipid peroxidation by increasing in vivo hepatic glutathione peroxidase activity (GPx) and reduced glutathione (GSH) levels in liver, with a dose-dependent reduction in TBA reactive substances [22]. Moreover, bioactive proteins recovered from ginseng have also been implicated in suppressing energy and lipid metabolism pathways by protection against cardiovascular ischemia-induced peroxidation [29]. Therefore, it is important to note that a plausible influence of feeding ginseng on broiler physiology and lipid metabolism was not the major contributing factor to reduce chemical lipid oxidation reactions in meats during refrigeration or frozen storage.

In the present study, we confirmed the findings of others that feeding broilers the vegetable-oil-based diet resulted in meat products that had a relatively higher proportion of PUFA, susceptible to oxidation reactions [30,31,32,33]. A decrease in the proportion of total PUFA in refrigerated and frozen stored meats has been formally attributed to losses in *n*-*6* PUFA, such as linoleic acid, and these losses were relatively greater in leg meat compared to breast meat [34]. Broilers fed on the vegetable-oil-based diets produced meat products with higher lipid hydroperoxide content than animal-fat-based diets in general, which agrees with the relatively higher total PUFA content and greater susceptibility for oxidation. Moreover, changes in total PUFA content of both leg and breast meats derived from birds fed vegetable-oil-based diet during short-term refrigerated storage were also affected by the inclusion of ginseng. The fact that broiler breast meats contained relatively lower lipid peroxide concentrations than corresponding leg meat samples points to known differences in potential prooxidants in these distinct muscle systems. Lipid oxidation occurring in breast meats is attributed to a relatively high phospholipid content, whereas heme proteins, including hemoglobin and myoglobin are more relevant in leg meats and have greater prooxidant potential than inorganic iron. Heme iron reacts with hydrogen peroxide to produce ferryl-myoglobin radicals that initiate lipid oxidation. We observed initial lipid hydroperoxide concentrations that reflected the extent of early primary stages of oxidation that increased in both refrigeration and frozen storage. In refrigeration storage, the lipid hydroperoxide level in ginseng-supplemented vegetable oil diet groups dropped after day 4. This temporal pattern refers to the relatively earlier transition from primary lipid hydroperoxides to subsequent generation of secondary products of lipid oxidation, such as MDA, or alternatively, complex reactions that result in hydroperoxide degradation during storage. The latter explanation is attributed to myoglobin degradation of hydroperoxides [35] or losses due to transformation with proteins and amino acids through Schiff base reactions [36,37,38].

The higher lipid hydroperoxide content which followed a temporal change during both refrigeration and frozen storage of broiler meats derived from birds fed on ginseng-containing diets appears to be in conflict with the recognized affinity of ginseng constituents that sequester free radicals [15,16]. Similar prooxidant findings have been reported, when garlic was added to chicken meat products before refrigeration storage [10]. This effect has also been shown with α-tocopherol, where the inhibition of hydroperoxide decomposition due to formation of ketodiene compounds was the underlying reason [39]. Moreover, in the absence of antioxidant, only a small proportion of initial lipid hydroperoxide was decomposed which corresponded to a corresponding increase in TBA values. Trolox, which traps alkoxyl radicals raised from linoleate hydroperoxides, converts them to stable hydroxy compounds by H-donation; a favored reaction compared to some degradation pathways. Thus, the potential prooxidant activity of ginseng may be misleading since it translates to the relative rate of lipid peroxide turnover at the initial stages of lipid oxidation. Lipid peroxides accumulate at first stages of oxidation reactions when the rate of formation exceeds the rate of decomposition; the reverse occurring at later stages of lipid oxidation [34]. For example, monomolecular decomposition predominates when concentration of lipid peroxides is initially low, whereas when the concentration of lipid peroxides increase, bimolecular decomposition occurs. The kinetic constant describing monomolecular decomposition of fatty acid autooxidation is lower than bimolecular decomposition. Our results show that this explanation was evident in broilers fed on vegetable oil diets containing a relatively higher PUFA content, which would increase susceptibility of initial peroxidation reactions [2]. The ingestion of ginseng in broilers fed this diet stabilized lipid hydroperoxides rather than contributing to degradation to secondary products of lipid oxidation, such as malonaldehyde. There is other evidence of this with ginsenosides Rb_1_ and Rg_1_ having properties to inhibit MDA production, induced by free radicals in liver and brain microsome preparations in vitro [40,41]. Although these same ginsenosides were present in the prong used in our studies, the fact that we could not recover ginsenosides in breast or leg meats from these birds weakens our conclusion. Notwithstanding this, however, others have reported recovering ginseng metabolites from the gut that reflected intestinal biotransformation while also retaining bioactive properties [26]. Further studies are required to determine if intestinal metabolites of ginseng are involved in lipid peroxidation reactions in poultry meats.

## 4. Materials and Methods

### 4.1. Dietary Composition

Broilers were raised in 30 floor pens, each pen holding 50 birds, and fed with one of six different grower diets for 42 days. Control broilers were fed with either a standard commercial animal fat (tallow) based (metabolic equivalents [ME], 3150 kcal/kg), or a vegetable-oil-based (Sungrown ^TM^; ME, 2926 kcal/g) grower diet, both of which contained 20% crude protein and added methionine + cysteine and lysine. Experimental diets were formulated to also include either 0.1% or 0.2% (*w*/*w*) of ginseng (*Panax quinquefolius* L.) prong powder in both the animal fat- and vegetable-oil-based formulations, respectively. The total ginsenoside content of the prong powder was 1.13% (*w*/*w*), and comprised of the major ginsenosides Rb_1_ (0.02%), Rb_2_ (0.05%), Rc (0.01%), Rd (0.22%), Re (0.2%), Rg_1_ (0.05%) *w*/*w*. A complete breakdown of the experimental diets is given in in Appendix A Table A1.

### 4.2. Poultry Meat Sample Preparation

All broilers were slaughtered at 42 days of age by electrical stunning followed by exsanguination and mechanically eviscerated. Both breast and leg samples were taken from air-chilled carcasses, packaged individually and placed in refrigerated storage until further processing occurred a minimum of 2 days after slaughter.

Experiment 1 consisted of a 10-day storage period at a refrigeration temperature set at 4 °C. Eight broilers fed with each diet were processed by manual cutting and breast and leg samples were stored in Ziplock freezer bags at 4 °C, until removed for chemical analysis of refrigerated storage. Storage. For analysis, breast and leg samples, respectively, were ground through a 0.4-cm plate on a Model 84142 Hobart grinder (Hobart Co., Troy, OH, USA) at 4 ± 1 °C. Minced samples were only prepared right before analysis of crude lipid and measures of oxidative stability were done daily.

Experiment 2 was a long-term frozen storage experiment conducted for up to 6 months in freezers set at −35 °C. Samples of breast and leg meats of broilers were collected as described above, and similar chemical analysis was done monthly. The processing procedures used for mincing poultry meat samples were identical to the short-term refrigerating experiment.

### 4.3. Crude Lipid Measurements

Minced samples (2.5 g) were homogenized using a Polytron PT 3000 (Kinematica^®^, Lucerne, Switzerland) for 30 s and added to flasks containing modified Folch’s solution (2:1 *v*/*v* chloroform:methanol; Fisher Scientific Co., Fair Lawn, NJ, USA) and 0.02% butylated hydroxytoluene (BHT; Sigma Chemical Co., St. Louis, MO, USA), to a final concentration of ~35 μg/mg fat [42]. Thereafter, the solution was passed through fluted Whatman #1 filter paper into 100-mL glass-stoppered graduated cylinders. Nonlipid substances were extracted by adding 10 mL of 0.88% (*w*/*v*) sodium chloride (Fisher Scientific) to the filtrate. A second extraction was conducted and the final volume (Vf) of the bottom lipid layer recorded. An aliquot of the bottom layer was transferred to dry, aluminum dishes of known weight and flushed with nitrogen gas. The dishes were weighed and placed in a desiccator overnight. Ratios of crude lipid (% *w*/*w*) were determined at the beginning and at the end of storage. Four replicate samples were measured in triplicate. The crude lipid content (%) was calculated as:

(1) $$\begin{array}{l} \mathrm{Crude}\;\mathrm{lipid}\;\mathrm{content}\;(\%)\\ =\frac{\mathrm{Vf}\;\times \;[\left(\mathrm{Weight}\;\mathrm{of}\;\mathrm{lipid}\;\mathrm{extract}\;\mathrm{in}\;\mathrm{aliquot}+\mathrm{dish}\right)-\;\mathrm{Weight}\;\mathrm{of}\;\mathrm{dish}}{\mathrm{Volume}\;\mathrm{of}\;\mathrm{aliquot}\;\times \;\mathrm{Original}\;\mathrm{weight}\;\mathrm{of}\;\mathrm{sample}}\;\times \;100\%\; \end{array}$$

### 4.4. Fatty Acid Measurements

Fatty acid profiles of the breast and leg meats were determined by gas chromatography-flame ion detection (GC-FID) [43]. Crude lipids derived from breast and leg meat samples were mixed with 5.0 mL of 0.5 N (*w*/*v*) potassium hydroxide in methanol (KOH-MeOH; both from Fisher Scientific), and then heated at 50 °C for one hour. Samples were cooled, suspended in 2.5 mL petroleum ether (Fisher Scientific) and the nonsaponifiable and ether layers removed by vacuum suction. Samples were treated with 0.1 mL of 0.4 M HCl (Fisher Scientific) and 5 mL of boron trifluoride-methanol (BF_3_-MeOH) for methylation. Derivatized fatty acid methyl esters (FAME) were recovered in hexane (Fisher Scientific) and sample FAME were separated and identified by comparing retention times with those of pure FAME standards (Sigma), using a GC-17A gas chromatograph (Shimadzu, Kyoto, Japan), equipped with a flame ion detector (FID) and an Omegawax^®^ 320 GC capillary column (30 m *×* 0.32 mm i.d.; 0.25 μm fused silica film thickness (Supelco Inc., Bellefonte, PA, USA). Analytical parameters included: flow rate of helium carrier gas, 113.0 mL/min; column flow rate, 1.1 mL/min; injected sample volume, 1 μL; split ratio, 100:1; velocity, 25.0 cm/s; total pressure, 66 kPa; temperatures of injector and detector, 250 °C and 260 °C, respectively; initial isothermal column temperature, 200 °C. Response factors (RF) corresponding to FAME were determined based on fatty acid profiles of standard FAME on GC chromatograms. Four crude lipid samples were methylated in duplicate. Fatty acid relative ratios (%) were determined from peak areas of individual fatty acids and the total peak area of all fatty acids using Class-VP chromatography automated software. The relative ratios (%) were determined according to:

(2) $$\mathrm{Individual}\;\mathrm{fatty}\;\mathrm{acid}\;\mathrm{content}\;\left(\%\right)=\frac{\mathrm{Individual}\;\mathrm{fatty}\;\mathrm{acid}\;\mathrm{peak}\;\mathrm{area}}{\mathrm{Total}\;\mathrm{fatty}\;\mathrm{acids}\;\mathrm{peak}\;\mathrm{area}}\;\times \;100\%\;\times \;\mathrm{RF}$$

### 4.5. Lipid Oxidation Measurements

Lipid oxidation was assessed by measuring both primary (e.g., lipid hydroperoxides using ferrous oxidation-xylenol orange (FOX) reagent [44]; and secondary products (e.g., malonaldehyde using (TBARS) reagent [45,46]. Four replicates of meats from broilers fed with each diet were measured in triplicate in the FOX and TBARS, analyses, respectively. FOX and TBARS measurements for long term storage were done at monthly intervals from the beginning (0 months equated to nonfrozen) to the end of a 6-month storage period.

#### 4.5.1. Primary Products of Lipid Oxidation

The FOX assay was used to measure concentration of lipid peroxides (ROOH) in broiler meats, a primary lipid oxidation product that oxidizes ferrous to ferric ion. To calibrate lipid peroxides, a hydrogen peroxide solution was used generate a calibration standard curve within a concentration range of 0–120 μM. We used a molar extinction coefficient of hydrogen peroxide at 240 nm of 43.6 M^−1^cm^−1^ [47]. One gram of minced meat sample was homogenized in 4 mL of propanol using a Polytron PT 3000 (Kinematica^®^, Bohemia, NY, USA). Samples were centrifuged for 10 min at 12,000× *g* in a Model 5402 centrifuge (Eppendorf) at room temperature (24 °C). Fresh pre-FOX reagent was prepared from xylenol orange (1 mM; Sigma) and 2.5 mM ferrous ammonium (Sigma) in 250 mM sodium sulfate (Fisher Scientific). The FOX reagent was prepared from a mixture of pre-FOX solution and methanol containing 4.4 mM BHT (1:9 *v*/*v*). Samples were reacted with fresh FOX reagent, and left for 30 min before absorbance was determined at 560 nm using a UV-160 spectrophotometer (Shimadzu) and quartz cuvettes to avoid disturbances caused by plastic deterioration. Four replicates of minced samples from broilers fed with each diet were assayed in triplicate. Absorbance at 560 nm was converted to lipid peroxide concentration [ROOH] (μM) according to the standard equation below:

(3) $$\left[\mathrm{ROOH}\right]{\;(\mu M)=\mathrm{Abs}}_{560\mathrm{nm}}\;\times \;133.6\;-\;1{.765\;(R}^{2}=0.996)$$

where:

Abs_560nm_ = absorbance measured at 560 nm

R^2^ = coefficient of determination (e.g., the square of Pearson product moment calculation coefficient from known hydrogen peroxide concentrations and corresponding absorbance measured at 560 nm)

#### 4.5.2. Thiobarbituric Acid Reactive Substances (TBARS) Assay

This assay measures the TBA-reactive malondialdehyde (MDA), a secondary lipid oxidation chromogen with a maximal absorbance wavelength of 532 nm. The assay was calibrated using a standard of 1,1,3,3-tetraethoxypropane (TEP; Sigma) in aqueous solution. Standard curves for TBARS were plotted in a straight line over the range of 0–10 μM. The TBARS concentration (μM) was calculated as:

(4) $$\left[\mathrm{TBARS}\right]{\;(\mu M)=\mathrm{Abs}}_{532\mathrm{nm}}\;\times \;0.1107+1{.765\;(R}^{2}=0.999)\;$$

where:

Abs_532nm_ = Absorbance measured at 532 nm

R^2^ = Coefficient of determination (e.g., the square of Pearson product moment correlation coefficient through data points in known-TEP concentrations and corresponding absorbance measured at 532 nm)

For TBARS analysis in meats, minced samples (2.5 g) were homogenized with 25 mL of 1.6% phosphoric acid (Fisher Scientific) in 20% trichloroacetic acid (TCA; Fisher Scientific) using a Polytron PT 3000 (Kinematica^®^). Filtrated samples were vortex-mixed with fresh TBA reagent (0.5% 2-thiobarbituric acid and 0.02% butylated hydroxytoluene in 0.025 M sodium hydroxide). The tubes were heated in boiling water bath for 15 min, cooled to room temperature and then read at an absorbance of 532 nm. Four replicates of meat samples from broilers fed with each diet were assayed in triplicate. Absorbance was converted into a TBA value (mg MDA/kg meat) using the following equations [48]:

(5) $${\mathrm{TBA}\;\mathrm{value}\;(\mathrm{mg}\;\mathrm{MDA}/\mathrm{kg}\;\mathrm{meat})=\mathrm{Abs}}_{532\mathrm{nm}}(\mathrm{Sample})\;\times \;K\;$$

(6) $$K=\frac{\mathrm{Standard}}{{\;\mathrm{Abs}}_{532\mathrm{nm}}\left(\mathrm{Standard}\right)}\;\times \;\mathrm{MW}\;\times \;\frac{10^{6}}{E}$$

where:

Abs_532nm_(Sample) = absorbance of sample at 532 nm

K = constant coefficient

Standard = number of moles TBAR standard (μmol in 2 mL)

Abs_532nm_(Standard) = absorbance of standard at 532 nm

MW = molecular weight (72.063 g/mol)

E = sample equivalent

A standard containing 2 *×* 10^−8^ mol of TEP/2mL (10 μM) was 1.074. The sample equivalent (E) was 0.1 g, in which 2.5 g of sample was diluted to 50.0 mL and 2.0 mL was analyzed. These constants resulted in a K value of 13.4. The TBA values were calculated by multiplying the absorbance by 13.4.

### 4.6. Statistical Analysis

Data were statistically analyzed using a one-way analysis of variance (ANOVA) with post hoc analysis done by Tukey’s test using MINITAB Version 12, Statistical Software (Minitab Inc., State College, PA, USA; 1998) according to Zar [49]. A two-way ANOVA was also performed to determine interactions of ginseng fortification level and storage time [49].

## 5. Conclusions

This study is the first to report that the use of ginseng prong, when added as an Appendix to broiler diets, was active at mitigating lipid peroxidation in poultry meats during cold storage. From our results, the antiperoxidant activity of ginseng appears to be related to the decomposition of lipid hydroperoxides, and production of secondary oxidation products, such as MDA. The free radical scavenging capacity formerly reported by others could also be involved in chain reactions that ultimately result in oxidation-related rancidity, contributing to loss of sensory and nutritional quality. Our findings indicated that controlling lipid peroxidation reactions in broiler meats can be achieved by the inclusion of dietary ginseng prong power fed to birds throughout their life cycle, when standard employment of cold temperatures during storage of meat products is practiced.

## Figures and Tables

**Table 1:** Crude lipid content (%) of breast and leg meat from broilers fed with diets containing animal fat or vegetable oil and supplemented with ginseng, stored at 4 °C ^1,2^.

**Diet/Storage (Days)**			
		**Breast meat**	
**Animal fat diet**	**A diet**	**B diet**	**C diet**
Day 0	1.25 ± 0.25 ^ax^	1.37 ± 0.32 ^ax^	1.66 ± 0.27 ^ay^
Day 10	1.29 ± 0.18 ^ax^	1.34 ± 0.14 ^ax^	1.41 ± 0.28 ^bx^
**Vegetable oil diet**	**D diet**	**E diet**	**F diet**
Day 0	1.49 ± 0.17 ^ax^	1.68 ± 0.36 ^ax^	1.59 ± 0.26 ^ax^
Day 10	1.20 ± 0.42 ^bx^	1.72 ± 0.37 ^ay^	1.56 ± 0.40 ^ay^
		**Leg meat**	
**Animal fat diet**	**A diet**	**B diet**	**C diet**
Day 0	4.32 ± 0.36 ^ax^	4.21 ± 0.47 ^ax^	5.56 ± 0.76 ^ay^
Day 10	4.40 ± 0.35 ^ax^	4.22 ± 0.44 ^ax^	5.23 ± 0.21 ^ax^
**Vegetable oil diet**	**D diet**	**E diet**	**F diet**
Day 0	3.75 ± 0.34 ^ax^	4.08 ± 0.44 ^ax^	3.96 ± 0.48 ^ax^
Day 10	3.57 ± 0.21 ^ax^	3.79 ± 0.40 ^ax^	3.50 ± 0.26 ^bx^

^1^ Data are expressed as means ± SD of 12 determinations (triplicate measurements of four replicates). Superscripts: ^a,b^ denotes significant differences (*p* < 0.05) among means corresponding to various storage durations within column; ^x,y^ denotes significant differences (*p* < 0.05) among means corresponding to various dietary supplements within row. ^2^ A diet = commercial animal-fat-based diet, with no ginseng; B diet = commercial animal-fat-based diet, with 1 × ginseng; C diet = commercial animal-fat-based diet, with 2 × ginseng; D diet = vegetable oil-based diet ^TM^, with no ginseng; E diet = vegetable-oil-based diet ^TM^, with 1 × ginseng; F diet = vegetable-oil-based diet ^TM^, with 2 × ginseng.

**Table 2:** Total PUFA content (%) in breast and leg meat from broilers fed with diets containing animal fat or vegetable oil and supplemented with ginseng stored at 4 °C ^1–3^.

**Diet/Storage (Days)**			
		**Breast meat**	
**Animal fat diet**	**A diet**	**B diet**	**C diet**
Day 0	19.61 ± 2.74 ^ax^	17.71 ± 1.65 ^ax^	18.33 ± 2.39 ^ax^
Day 10	16.97 ± 2.40 ^ax^	16.47 ± 1.45 ^ax^	16.56 ± 0.74 ^ax^
**Vegetable oil diet**	**D diet**	**E diet**	**F diet**
Day 0	20.67 ± 1.47 ^bx^	20.01 ± 2.00 ^ax^	19.35 ± 1.84 ^ax^
Day 10	18.18 ± 1.10 ^ay^	16.55 ± 2.05 ^bx^	16.39 ± 1.80 ^bx^
		**Leg meat**	
**Animal fat diet**	**A diet**	**B diet**	**C diet**
Day 0	15.86 ± 1.18 ^ax^	15.85 ± 0.79 ^ax^	14.87 ± 0.59 ^ax^
Day 10	15.19 ± 0.75 ^ax^	15.38 ± 0.85 ^ax^	14.26 ± 0.82 ^ax^
**Vegetable oil diet**	**D diet**	**E diet**	**F diet**
Day 0	21.04 ± 1.80 ^bx^	19.35 ± 2.57 ^bx^	18.44 ± 1.78 ^bx^
Day 10	19.20 ± 0.95 ^ay^	16.64 ± 1.50 ^ax^	15.94 ± 2.01 ^ax^

^1^ Total PUFAs = Sum of n-6 and n-3 PUFAs. ^2^ Data are expressed as means ± SD of eight determinations (single measurements of two derivatized samples of four extracts). Superscripts: ^a,b^ denotes significant differences (*p* < 0.05) among means corresponding to various storage durations within column; ^x,y^ denotes significant differences (*p* < 0.05) among means corresponding to various dietary supplements within row. ^3^ A diet = commercial animal-fat-based diet, with no ginseng; B diet = commercial animal-fat-based diet, with 1 × ginseng; C diet = commercial animal-fat-based diet, with 2 × ginseng; D diet = vegetable-oil-based diet ^TM^, with no ginseng; E diet = vegetable-oil-based diet ^TM^, with 1 × ginseng; F diet = vegetable-oil-based diet ^TM^, with 2 × ginseng.

**Table 3:** Lipid hydroperoxide values (μM) of breast and leg meats stored at 4 °C from broilers fed on diets containing animal fat or vegetable oil and supplemented with ginseng ^1−3^.

**Diet/Storage (Days)**			
		**Breast meat**	
**Animal fat diet**	**A diet**	**B diet**	**C diet**
Day 0	ND ^ax^	ND ^ax^	0.02 ± 0.01 ^ay^
Day 2	3.63 ± 0.51 ^bx^	3.63 ± 0.38 ^bx^	4.51 ± 0.55 ^by^
Day 4	5.04 ± 0.84 ^cx^	6.32 ± 0.52 ^cy^	6.69 ± 0.66 ^cy^
Day 7	8.28 ± 0.67 ^dx^	8.27 ± 0.54 ^dx^	10.06 ± 0.77 ^dy^
Day 10	20.40 ± 1.84 ^ey^	12.62 ± 1.10 ^ex^	24.08 ± 1.51 ^ez^
**Vegetable oil diet**	**D diet**	**E diet**	**F diet**
Day 0	8.18 ± 0.33 ^ax^	7.25 ± 0.60 ^ax^	9.19 ± 0.45 ^ax^
Day 2	27.20 ± 1.31 ^bx^	61.89 ± 2.03 ^cy^	85.79 ± 2.88 ^cz^
Day 4	30.96 ± 1.43 ^cx^	64.65 ± 2.60 ^cy^	102.32 ± 4.98 ^dz^
Day 7	57.52 ± 7.63 ^dy^	37.87 ± 2.59 ^bx^	61.87 ± 2.41 ^by^
Day 10	53.48 ± 4.30 ^dx^	46.73 ± 6.86 ^bx^	54.86 ± 7.36 ^bx^
		**Leg meat**	
**Animal fat diet**	**A diet**	**B diet**	**C diet**
Day 0	1.59 ± 0.15 ^ax^	3.62 ± 0.26 ^az^	2.31 ± 0.12 ^ay^
Day 2	39.29 ± 2.60 ^bx^	39.37 ± 4.44 ^bx^	45.75 ± 3.99 ^bx^
Day 4	49.00 ± 5.17 ^bcx^	62.40 ± 3.78 ^cy^	67.30 ± 7.41 ^cy^
Day 7	54.51 ± 3.54 ^cdx^	69.11 ± 5.66 ^cy^	81.25 ± 6.83 ^dz^
Day 10	64.98 ± 8.16 ^dx^	79.23 ± 7.39 ^cx^	94.41 ± 6.57 ^dy^
**Vegetable oil diet**	**D diet**	**E diet**	**F diet**
Day 0	79.7 ± 6.9 ^ax^	122.5 ± 18.1 ^ay^	126.3 ± 28.1 ^ay^
Day 2	176.4 ± 13.9 ^cx^	202.8 ± 36.0 ^cy^	219.7 ± 23.1 ^bz^
Day 4	183.6 ± 19.4 ^cx^	214.7 ± 18.2 ^cy^	203.8 ± 23.0 ^by^
Day 7	105.6 ± 18.2 ^bx^	181.9 ± 28.1 ^bz^	144.3 ± 18.7 ^ay^
Day 10	87.8 ± 11.0 ^ax^	157.2 ± 19.6 ^ay^	153.5 ± 11.2 ^ay^

^1^ Data are expressed as means ± SD of 12 determinations (triplicate measurements of four replicates). ND, not detectable (<0.01 μM). ^2^ Superscripts: ^a,b,c,d,e^ denotes significant differences (*p* < 0.05) among means corresponding to various storage durations within column; ^x,y,z^ denotes significant differences (*p* < 0.05) among means corresponding to various dietary supplements within row. ^3^ A diet = commercial animal-fat-based diet, with no ginseng; B diet = commercial animal-fat-based diet, with 1 × ginseng; C diet = commercial animal-fat-based diet, with 2 × ginseng; D diet = vegetable-oil-based diet ^TM^, with no ginseng; E diet = vegetable-oil-based diet ^TM^, with 1 × ginseng; F diet = vegetable-oil-based diet ^TM^, with 2 × ginseng.

**Table 4:** TBA values (mg MDA/kg meat) of breast and leg meat stored at 4 °C, from broilers fed on diets containing animal fat or vegetable oil and supplemented with ginseng ^1−3^.

**Diet/Storage (Days)**			
		**Breast meat**	
**Animal fat diet**	**A diet**	**B diet**	**C diet**
Day 0	2.37 ± 0.09 ^a^^z^	0.53 ± 0.04 ^ay^	0.27 ± 0.01 ^ax^
Day 2	3.92 ± 0.14 ^bz^	2.34 ± 0.05 ^by^	1.31 ± 0.06 ^bx^
Day 4	4.36 ± 0.19 ^cz^	2.58 ± 0.08 ^dy^	2.38 ± 0.11 ^cx^
Day 7	5.17 ± 0.31 ^dz^	2.47 ± 0.12 ^cy^	2.52 ± 0.01 ^dx^
Day 10	3.40 ± 0.19 ^by^	2.90 ± 0.04 ^ex^	2.79 ± 0.09 ^ex^
**Vegetable oil diet**	**D diet**	**E diet**	**F diet**
Day 0	2.09 ± 0.06 ^az^	1.38 ± 0.12 ^ay^	1.14 ± 0.05 ^ax^
Day 2	7.17 ± 0.28 ^bcz^	2.79 ± 0.08 ^bx^	4.01 ± 0.25 ^by^
Day 4	8.27 ± 0.24 ^cy^	3.89 ± 0.10 ^bcx^	3.93 ± 0.24 ^bx^
Day 7	6.69 ± 0.24 ^bz^	5.10 ± 0.30 ^cx^	6.39 ± 0.19 ^cy^
Day 10	5.56 ± 0.34 ^bx^	5.30 ± 0.41 ^cx^	6.78 ± 0.15 ^cy^
		**Leg meat**	
**Animal fat diet**	**A diet**	**B diet**	**C diet**
Day 0	2.16 ± 0.07 ^az^	1.39 ± 0.06 ^ay^	1.19 ± 0.04 ^ax^
Day 2	4.85 ± 0.05 ^bz^	3.67 ± 0.11 ^by^	2.41 ± 0.09 ^bcx^
Day 4	5.48 ± 0.17 ^cz^	3.65 ± 0.22 ^by^	2.39 ± 0.11 ^bx^
Day 7	5.61 ± 0.07 ^cz^	4.14 ± 0.12 ^cy^	2.51 ± 0.08 ^cx^
Day 10	2.88 ± 0.05 ^ay^	3.88 ± 0.25 ^bz^	2.51 ± 0.10 ^cx^
**Vegetable oil diet**	**D diet**	**E diet**	**F diet**
Day 0	4.08 ± 0.06 ^az^	3.68 ± 0.05 ^by^	2.18 ± 0.09 ^ax^
Day 2	6.89 ± 0.36 ^bz^	3.20 ± 0.19 ^ax^	3.73 ± 0.20 ^by^
Day 4	7.99 ± 0.45 ^cy^	3.82 ± 0.13 ^cx^	3.77 ± 0.19 ^bx^
Day 7	7.92 ± 0.42 ^cz^	6.38 ± 0.25 ^dy^	5.51 ± 0.37 ^cx^
Day 10	9.56 ± 0.64 ^dz^	7.71 ± 0.40 ^ey^	6.71 ± 0.37 ^dx^

^1^ Data are expressed as means ± SD of 12 determinations (triplicate measurements of four replicates). ^2^ Superscripts: ^a,b,c,d,e^ denotes significant differences (*p* < 0.05) among means corresponding to various storage durations within column; ^x,y,z^ denotes significant differences (*p* < 0.05) among means corresponding to various dietary supplements within row. ^3^ A diet = commercial animal-fat-based diet, with no ginseng; B diet = commercial animal-fat-based diet, with 1 × ginseng; C diet = commercial animal-fat-based diet, with 2 × ginseng; D diet = vegetable-oil-based diet ^TM^, with no ginseng; E diet = vegetable-oil-based diet ^TM^, with 1 × ginseng; F diet = vegetable-oil-based diet ^TM^, with 2 × ginseng.

**Table 5:** Crude lipid content (%) of breast and leg meats with long-term frozen (−18 °C) from broilers fed on diets containing animal fat or vegetable oil and supplemented with ginseng ^1−3^.

**Diet/Storage (Months)**			
		**Breast meat**	
**Animal fat diet**	**A diet**	**B diet**	**C diet**
Month 0	1.46 ± 0.21 ^bx^	1.49 ± 0.20 ^bx^	1.64 ± 0.35 ^by^
Month 2	1.10 ± 0.45 ^ax^	1.29 ± 0.30 ^ax^	1.45 ± 0.25 ^by^
Month 4	1.22 ± 0.35 ^ax^	1.20 ± 0.10 ^ax^	1.17 ± 033 ^ax^
Month 6	1.03 ± 0.43 ^ax^	1.27 ± 0.25 ^ay^	1.15 ± 0.45 ^ay^
**Vegetable oil diet**	**D diet**	**E diet**	**F diet**
Month 0	1.36 ± 0.84 ^bx^	1.51 ± 0.93 ^ay^	1.71 ± 0.50 ^by^
Month 2	1.20 ± 0.45 ^ax^	1.40 ± 0.27 ^ay^	1.66 ± 0.17 ^bz^
Month 4	1.26 ± 0.32 ^ax^	1.42 ± 0.85 ^ay^	1.70 ± 0.38 ^bz^
Month 6	1.24 ± 0.17 ^ax^	1.39 ± 0.23 ^ay^	1.40 ± 0.13 ^ay^
		**Leg meat**	
**Animal fat diet**	**A diet**	**B diet**	**C diet**
Month 0	4.23 ± 0.32 ^ax^	4.77 ± 0.25 ^ax^	4.84 ± 0.22 ^bx^
Month 2	4.17 ± 0.41 ^ax^	4.20 ± 0.33 ^ax^	4.29 ± 0.49 ^ax^
Month 4	4.08 ± 0.30 ^ax^	4.48 ± 0.34 ^ay^	4.28 ± 0.24 ^axy^
Month 6	4.11 ± 0.24 ^ax^	4.33 ± 0.40 ^ax^	4.20 ± 0.36 ^ax^
**Vegetable oil diet**	**D diet**	**E diet**	**F diet**
Month 0	4.24 ± 0.33 ^bx^	4.46 ± 0.34 ^bx^	4.79 ± 0.28 ^by^
Month 2	4.19 ± 0.14 ^bx^	4.22 ± 0.68 ^bx^	4.40 ± 0.20 ^abx^
Month 4	4.05 ± 0.44 ^ax^	3.92 ± 0.44 ^ax^	4.16 ± 0.13 ^ax^
Month 6	4.12 ± 0.60 ^bx^	3.93 ± 0.42 ^ax^	3.96 ± 0.46 ^ax^

^1^ Data are expressed as means ± SD of 12 determinations (triplicate measurements of four replicates). ^2^ Superscripts: ^a,b^ denotes significant differences (*p* < 0.05) among means corresponding to various storage durations within column; ^x,y,z^ denotes significant differences (*p* < 0.05) among means corresponding to various dietary supplements within row. ^3^ A diet = commercial animal-fat-based diet, with no ginseng; B diet = commercial animal-fat-based diet, with 1 × ginseng; C diet = commercial animal-fat-based diet, with 2 × ginseng; D diet = vegetable-oil-based diet ^TM^, with no ginseng; E diet = vegetable-oil-based diet ^TM^, with 1 × ginseng; F diet = vegetable-oil-based diet ^TM^, with 2 × ginseng.

**Table 6:** Lipid hydroperoxide content (μM) of breast and leg meat stored at −18 °C, from broilers fed on diets containing animal fat or vegetable oil and supplemented with ginseng ^1−3^.

**Diet/Storage (Months)**			
		**Breast meat**	
**Animal fat diet**	**A diet**	**B diet**	**C diet**
Month 0	1.53 ± 0.17 ^ay^	1.56 ± 0.16 ^ay^	1.22 ± 0.21 ^ax^
Month 1	3.43 ± 0.27 ^ay^	2.26 ± 0.29 ^ax^	2.06 ± 0.22 ^ax^
Month 2	21.71 ± 3.39 ^bx^	57.12 ± 9.48 ^cz^	45.21 ± 5.31 ^c^^y^
Month 4	28.94 ± 3.35 ^bx^	35.62 ± 3.73 ^by^	29.01 ± 1.76 ^bx^
Month 6	2.49 ± 0.89 ^ax^	1.58 ± 0.65 ^ax^	1.38 ± 0.85 ^ax^
**Vegetable oil diet**	**D diet**	**E diet**	**F diet**
Month 0	2.52 ± 0.97 ^ax^	2.69 ± 0.66 ^ax^	1.99 ± 0.55 ^ax^
Month 1	3.55 ± 0.25 ^ay^	2.47 ± 0.21 ^ax^	2.64 ± 0.29 ^ax^
Month 2	29.12 ± 5.78 ^bx^	35.92 ± 2.81 ^by^	43.89 ± 7.37 ^by^
Month 4	49.48 ± 1.57 ^cx^	42.89 ± 4.22 ^cx^	39.93 ± 3.55 ^bx^
Month 6	1.26 ± 0.24 ^ay^	0.17 ± 0.11 ^ax^	0.18 ± 0.09 ^ax^
		**Leg meat**	
**Animal fat diet**	**A diet**	**B diet**	**C diet**
Month 0	1.66 ± 0.13 ^ax^	1.68 ± 0.10 ^ax^	1.71 ± 0.12 ^ax^
Month 1	2.41 ± 0.26 ^by^	2.13 ± 0.24 ^by^	1.75 ± 0.23 ^ax^
Month 2	17.03 ± 2.01 ^cx^	18.44 ± 1.58 ^cx^	21.24 ± 2.16 ^cx^
Month 4	55.87 ± 12.9 ^ez^	39.63 ± 1.43 ^ey^	28.18 ± 2.37 ^dx^
Month 6	30.89 ± 4.2 ^dy^	27.04 ± 3.36 ^dy^	16.82 ± 3.12 ^bx^
**Vegetable oil diet**	**D diet**	**E diet**	**F diet**
Month 0	5.47 ± 1.69 ^ay^	3.39 ± 1.50 ^ax^	1.95 ± 0.17 ^ax^
Month 1	7.10 ± 0.38 ^ay^	5.74 ± 0.50 ^ax^	5.01 ± 0.43 ^bx^
Month 2	21.23 ± 2.77 ^bx^	23.74 ± 1.60 ^bx^	24.56 ± 2.20 ^cx^
Month 4	58.74 ± 3.99 ^dx^	77.56 ± 4.29 ^cy^	83.92 ± 9.32 ^dy^
Month 6	38.94 ± 1.08 ^cy^	26.04 ± 2.23 ^bx^	20.80 ± 2.22 ^cx^

^1^ Data are expressed as means ± SD of 12 determinations (triplicate measurements of four replicates). ^2^ Superscripts: ^a,b,c,d,e^ denotes significant differences (*p* < 0.05) among means corresponding to various storage durations within column; ^x,y,z^ denotes significant differences (*p* < 0.05) among means corresponding to various dietary supplements within row. ^3^ A diet = Commercial animal-fat-based diet, with no ginseng; B diet = Commercial animal-fat-based diet, with 1 × ginseng; C diet = Commercial animal-fat-based diet, with 2 × ginseng; D diet = Vegetable-oil-based diet ^TM^, with no ginseng; E diet = Vegetable-oil-based diet ^TM^, with 1 × ginseng; F diet = Vegetable-oil-based diet ^TM^, with 2 × ginseng.

**Table 7:** TBA values (mg MDA/kg meat) of breast and leg meat stored at −18 °C, from broilers fed on diets containing animal fat or vegetable oil and supplemented with ginseng ^1−3^.

**Diet/Storage (Months)**			
		**Breast meat**	
**Animal fat diet**	**A diet**	**B diet**	**C diet**
Month 0	0.22 ± 0.01 ^ax^	0.20 ± 0.01 ^ax^	0.20 ± 0.01 ^ax^
Month 1	0.73 ± 0.03 ^by^	0.37 ± 0.01 ^bx^	0.30 ± 0.01 ^bx^
Month 2	1.54 ± 0.07 ^cy^	0.74 ± 0.03 ^cx^	0.91 ± 0.05 ^cx^
Month 4	2.64 ± 0.12 ^dy^	0.76 ± 0.05 ^cx^	0.68 ± 0.04 ^cx^
Month 6	0.45 ± 0.02 ^ax^	0.43 ± 0.03 ^bx^	0.43 ± 0.02 ^bx^
**Vegetable oil diet**	**D diet**	**E diet**	**F diet**
Month 0	0.25 ± 0.01 ^ay^	0.26 ± 0.01 ^ay^	0.18 ± 0.01 ^ax^
Month 1	0.36 ± 0.02 ^by^	0.28 ± 0.01 ^ax^	0.26 ± 0.02 ^bx^
Month 2	0.88 ± 0.04 ^cy^	0.83 ± 0.03 ^by^	0.21 ± 0.02 ^ax^
Month 4	2.63 ± 0.10 ^dy^	1.04 ± 0.04 ^cx^	0.98 ± 0.05 ^dx^
Month 6	0.89 ± 0.03 ^cy^	0.44 ± 0.02 ^abx^	0.41 ± 0.04 ^cx^
		**Leg meat**	
**Animal fat diet**	**A diet**	**B diet**	**C diet**
Month 0	0.34 ± 0.01 ^ay^	0.23 ± 0.01 ^ax^	0.20 ± 0.01 ^ax^
Month 1	1.47 ± 0.02 ^by^	0.33 ± 0.03 ^bx^	0.29 ± 0.02 ^ax^
Month 2	1.55 ± 0.09 ^bz^	0.90 ± 0.05 ^cx^	1.18 ± 0.04 ^by^
Month 4	2.27 ± 0.04 ^cz^	1.67 ± 0.09 ^dy^	0.99 ± 0.06 ^xz^
Month 6	0.91 ± 0.06 ^by^	0.75 ± 0.04 ^cx^	0.86 ± 0.10 ^by^
**Vegetable oil diet**	**D diet**	**E diet**	**F diet**
Month 0	0.37 ± 0.01 ^ax^	0.36 ± 0.02 ^ax^	0.37 ± 0.01 ^ax^
Month 1	0.45 ± 0.03 ^ay^	0.48 ± 0.03 ^ay^	0.38 ± 0.03 ^ax^
Month 2	0.83 ± 0.04 ^by^	0.84 ± 0.13 ^by^	0.37 ± 0.03 ^ax^
Month 4	2.76 ± 0.12 ^dx^	2.36 ± 0.12 ^cx^	2.48 ± 0.19 ^cx^
Month 6	1.10 ± 0.08 ^cy^	0.86 ± 0.05 ^bx^	0.76 ± 0.04 ^bx^

^1^ Data are expressed as means ± SD of 12 determinations (triplicate measurements of four replicates). ^2^ Superscripts: ^a,b,c,d^ denotes significant differences (*p* < 0.05) among means corresponding to various storage durations within column; ^x,y,z^ denotes significant differences (*p* < 0.05) among means corresponding to various dietary supplements within row. ^3^ A diet = commercial animal-fat-based diet, with no ginseng; B diet = commercial animal-fat-based diet, with 1 × ginseng; C diet = commercial animal-fat-based diet, with 2 × ginseng; D diet = vegetable-oil-based diet ^TM^, with no ginseng; E diet = vegetable-oil-based diet ^TM^, with 1 × ginseng; F diet = vegetable-oil-based diet ^TM^, with 2 × ginseng.

## Data Availability

Not Applicable.

## Sample Availability

Not Available.

## Appendix A

**Table A1:** Proximate analysis and composition of experimental basal diets ^1−6^.

Nutrient	Animal-Fat-Based Diet		Vegetable-Oil-Based Diet ^2^	
	Starter ^3^	Grower ^3^	Starter ^3^	Grower ^3^
Crude Protein (%)	23.00	20.00	22.80	21.70
Methionine + Cysteine (%)	0.83	0.70	0.83	0.80
Lysine (%)	1.16	1.00	1.13	1.02
Fat (%)	4.10	4.60	4.57	4.45
Fiber (%)	2.80	2.70	3.07	3.02
Calcium (%)	0.95	0.95	1.00	1.01
Phosphorus, total (%)	0.81	0.72	0.80	0.75
Phosphorus, available (%)	0.45	0.40	0.50	0.49
Sodium (%)	0.17	0.17	0.22	0.20
Metabolizable Energy (kcal/kg)	3075	3150	2924	2926

^1^ Data are provided by Canadian Poultry Consultants Ltd., 30325 Canary Court, Abbotsford, BC, Canada V4X 2N4. ^2^ Vegetable-oil based diet (Sungrown^TM^) was formulated and provided by W.R. Cox, Animal Health Research Services, Chilliwack, BC, Canada V2P 7 × 5^3^. ^3^ Starter diets were used to feed chicken broilers from 0–3 weeks; grower diets were used to feed chicken broilers from 4–6 weeks. Birds slaughtered at 42 days of age. ^4^ North American ginseng (*Panax quinquefolius* L.) prong, supplied from Chai-Na-Ta Corporation, had a total ginsenoside content of 1.13% (*w*/*w*). The ginsenoside percentage composition (*w*/*w*) was as follows: Rb_1_ (0.02%); Rb_2_ (0.05%); Rc (0.01%); Rd (0.22%); Re (0.20%); Rg_1_ (0.05%); and other total major ginsenosides (0.58%).

The results were provided by Celex Laboratories Inc., Atholville Industrial Mall, 77 Comeau Street, P.O. Box 428, Atholville, New Brunswick, Canada E0K 1A0.

**Table A2:** Initial carcass quality measurements for breast and leg meats derived from broilers fed on diets containing animal fat or vegetable oil and supplemented with ginseng ^1−3^.

		**Diets**	
**Quality Parameter**		**Breast Meat**	
**Animal fat diets**	**A diet**	**B diet**	**C diet**
pH	5.85 ± 0.05 ^x^	5.91 ± 0.140 ^x^	5.80 ± 0.04 ^x^
Ash (%)	1.34 ± 0.01 ^x^	1.31 ± 0.01 ^x^	1.33 ± 0.06 ^x^
Moisture (%)	71.78 ± 0.72 ^x^	71.47 ± 0.75 ^x^	70.27 ± 0.78 ^y^
HunterLab L*	44.77 ± 2.01 ^x^	36.69 ± 3.29 ^y^	38.98 ± 2.25 ^y^
HunterLab a*	6.10 ± 1.04 ^x^	6.90 ± 0.74 ^x^	6.49 ± 0.01 ^x^
HunterLab b*	12.20 ± 0.19 ^x^	9.33 ± 0.93 ^y^	9.55 ± 0.89 ^y^
**Vegetable oil diets**	**D diet**	**E diet**	**F diet**
pH	5.91 ± 0.09 ^x^	5.88 ± 0.08 ^x^	5.97 ± 0.08 ^x^
Ash (%)	1.24 ± 0.05 ^x^	1.27 ± 0.04 ^x^	1.28 ± 0.03 ^x^
Moisture (%)	70.94 ± 0.64 ^x^	71.48 ± 1.85 ^x^	71.10 ± 0.60 ^x^
HunterLab L*	32.13 ± 1.49 ^x^	31.78 ± 1.18 ^y^	30.38 ± 1.32 ^y^
HunterLab a*	11.06 ± 1.03 ^x^	12.20 ± 0.84 ^y^	12.42 ± 0.74 ^y^
HunterLab b*	12.24 ± 0.56 ^x^	12.45 ± 0.64 ^x^	12.58 ± 0.40 ^x^
		**Leg meat**	
**Animal fat diets**	**A diet**	**B diet**	**C diet**
pH	6.33 ± 0.09 ^x^	6.29 ± 0.08 ^x^	6.21 ± 0.04 ^x^
Ash (%)	1.08 ± 0.05 ^x^	1.06 ± 0.11 ^x^	1.19 ± 0.07 ^y^
Moisture (%)	70.37 ± 1.21 ^x^	69.78 ± 1.96 ^x^	68.27 ± 2.05 ^x^
HunterLab L*	40.33 ± 1.27 ^x^	36.70 ± 3.27 ^x^	41.54 ± 2.76 ^x^
HunterLab a*	10.78 ± 0.53 ^x^	10.73 ± 0.44 ^x^	11.13 ± 0.49 ^x^
HunterLab b*	11.98 ± 0.49 ^x^	8.55 ± 0.74 ^y^	10.90 ± 0.85 ^x^
**Vegetable oil diets**	**D diet**	**E diet**	**F diet**
pH	6.31 ± 0.06 ^x^	6.38 ± 0.09 ^x^	6.44 ± 0.05 ^y^
Ash (%)	1.13 ± 0.04 ^x^	1.21 ± 0.04 ^y^	1.27 ± 0.06 ^y^
Moisture (%)	67.67 ± 3.25 ^x^	68.83 ± 2.54 ^x^	69.71 ± 1.22 ^x^
HunterLab L*	34.50 ± 2.55 ^x^	34.32 ± 2.44 ^y^	34.91 ± 1.44 ^x^
HunterLab a*	11.08 ± 1.18 ^x^	13.80 ± 1.49 ^y^	12.53 ± 1.10 ^y^
HunterLab b*	12.71 ± 0.72 ^x^	13.24 ± 0.55 ^x^	13.44 ± 0.64 ^x^

^1^ Data are expressed as means ± SD of 12 determinations (triplicate measurements of four replicates). ND, not detectable (<0.01 μM). ^2^ Superscripts: ^x,y^ denotes significant differences (*p* < 0.05) among means corresponding to various dietary supplements within row. ^3^ A diet = commercial animal-fat-based diet, with no ginseng; B diet = commercial animal-fat-based diet, with 1 × ginseng; C diet = commercial animal-fat-based diet, with 2 × ginseng; D diet = vegetable-oil-based diet ^TM^, with no ginseng; E diet = vegetable-oil-based diet ^TM^, with 1 × ginseng; F diet = vegetable-oil-based diet ^TM^, with 2 × ginseng.
